# Mixed‐species groups of Serengeti grazers: a test of the stress gradient hypothesis

**DOI:** 10.1002/ecy.3163

**Published:** 2020-09-09

**Authors:** Lydia Beaudrot, Meredith S. Palmer, T. Michael Anderson, Craig Packer

**Affiliations:** ^1^ BioSciences Department Program in Ecology & Evolutionary Biology Rice University W100 George R. Brown Hall, 6100 Main Street, MS‐140 Houston Texas 77005 USA; ^2^ Department of Ecology & Evolutionary Biology Princeton University Princeton New Jersey 08544 USA; ^3^ Department of Biology Wake Forest University 1834 Wake Forest Drive Winston‐Salem Northern California 27109 USA; ^4^ Department of Ecology, Evolution and Behavior University of Minnesota St. Paul Minnesota 55108 USA

**Keywords:** associational defense, facilitation, group defense, habitat amelioration, heterospecific group, interspecific group, landscape of fear, polyspecific association, predation

## Abstract

Understanding the role of species interactions within communities is a central focus of ecology. A key challenge is to understand variation in species interactions along environmental gradients. The *stress gradient hypothesis* posits that positive interactions increase and competitive interactions decrease with increasing consumer pressure or environmental stress. This hypothesis has received extensive attention in plant community ecology, but only a handful of tests in animals. Furthermore, few empirical studies have examined multiple co‐occurring stressors. Here we test predictions of the stress gradient hypothesis using the occurrence of mixed‐species groups in six common grazing ungulate species within the Serengeti‐Mara ecosystem. We use mixed‐species groups as a proxy for potential positive interactions because they may enhance protection from predators or increase access to high‐quality forage. Alternatively, competition for resources may limit the formation of mixed‐species groups. Using more than 115,000 camera trap observations collected over 5 yr, we found that mixed‐species groups were more likely to occur in risky areas (i.e., areas closer to lion vantage points and in woodland habitat where lions hunt preferentially) and during time periods when resource levels were high. These results are consistent with the interpretation that stress from high predation risk may contribute to the formation of mixed‐species groups, but that competition for resources may prevent their formation when food availability is low. Our results are consistent with support for the stress gradient hypothesis in animals along a consumer pressure gradient while identifying the potential influence of a co‐occurring stressor, thus providing a link between research in plant community ecology on the stress gradient hypothesis, and research in animal ecology on trade‐offs between foraging and risk in landscapes of fear.

## Introduction

Understanding the role of species interactions within communities is a central focus of ecology. While there is a long history of research on competition (Elton 1946, Diamond 1975), a focus on positive interactions largely began at the start of this century (Stachowicz 2001, Bruno et al. 2003). Positive interactions between species are those in which one or both species benefit from an interaction and neither species is harmed. The outcomes of species interactions are not necessarily fixed as positive or negative, but can vary over space and time (Thompson 1999, van Ommeren and Whitham 2002) and can depend on environmental conditions (Dangles et al. 2018). A key challenge in ecology, therefore, is to identify the conditions under which positive and negative species interactions are more likely to occur.

The *stress gradient hypothesis* posits that positive interactions increase and competitive interactions decrease as consumer pressure or environmental stress increase (Bertness and Callaway 1994). For example, an organism faced with a high risk of being eaten may form an association as a defense against consumers, which is often referred to as an associational defense for plants or group defense among animals. Environmental stress from physical or abiotic conditions may result in species interactions that decrease stress by improving habitat conditions. For example, an organism faced with stress from low food availability could increase intake by copying foraging locations (Krebs 1973) or avoiding previously exploited areas (Beauchamp and Ruxton 2005). Thus, positive interactions among species are expected to be more common when consumer pressure or environmental stress is high. The stress gradient hypothesis has received extensive attention in plant community ecology (>700 studies; He et al. 2013). Its applicability to animal communities, however, has only recently been tested (e.g., Barrio et al. 2012, Dangles et al. 2018).

Since its formulation, considerable research in plant ecology has resulted in further refinement of the original stress gradient hypothesis. An important focus of current debate concerns the generality of the hypothesis, including the extent to which the hypothesis is applicable to less studied species, such as mobile (as opposed to sessile) animals, and understudied ecosystems, including the tropics (He and Bertness 2014). One approach for testing the stress gradient hypothesis in mobile animals is to evaluate the conditions under which mixed‐species groups form (Kamilar and Beaudrot 2013). Mixed‐species groups occur when one or more individuals of a group‐living species join a group of another species irrespective of concentrated resources, such as permanent water sources (Stensland et al. 2003). Mixed‐species groups are expected to form when they benefit individuals of one or more participating species and thus provide the potential for positive species interactions. Among vertebrates, mixed‐species groups occur in birds, fish, and mammals; among mammals, they occur in primates, cetaceans, and ungulates (Shaw 1962, Stensland et al. 2003, Sridhar et al. 2009), yet to our knowledge, no studies have used mixed‐species groups to test the stress gradient hypothesis.

Mixed‐species groups may form because individuals seek protection from predators. Similar to the advantages of forming single‐species groups, benefits from mixed‐species groups can result from multiple mechanisms (Terborgh 1990, Fryxell 1991). For example, mixed‐species groups may reduce predation risk because a given individual is less likely to be preyed upon in a group than when alone (i.e., the dilution effect; Foster and Treherne 1981). The movement of multiple individuals can confuse a predator and reduce the likelihood of a successful attack (Miller 1922). More individuals can also lead to better detection of predators due to increased vigilance (Pulliam 1973). Importantly, mixed‐species groups may provide additional advantages that differ from the advantages of single‐species groups, such as when the second species is better at detecting predators or uses a different sensory modality than the first species (Schmitt et al. 2016). Grouping behavior in both single species and mixed‐species groups may be selected for because grouping can allow individuals to spend less time on vigilance for predators and more time on other behaviors, such as foraging (Schmitt et al. 2014).

Individuals in mixed‐species groups may benefit from increased access to resources, such as dropped food or flushed prey (Heymann and Hsia 2015). Specifically in large mammalian herbivores, smaller‐bodied animals that require higher quality food sources and graze more selectively may benefit from foraging alongside larger, less selective grazers if grazing by the larger animals increases their ability to feed on the highest quality plant tissues, such as leaf and leaf sheath (Fryxell et al. 2005). On the other hand, increasing the total number of individuals in a group can increase competition for food (Schoener 1971), which may decrease the occurrence of mixed‐species groups when resources are scarce.

The Serengeti is an excellent natural system in which to test the stress gradient hypothesis using mixed‐species groups for several reasons. First, the Serengeti contains a hyper‐diverse ungulate community in which multiple types of mixed‐species groups among grazers have been observed (Sinclair 1985, Fitzgibbon 1990, Kiffner et al. 2014). Second, the apex predator in the system, the lion (*Panthera leo*), feeds predominantly on group‐living prey rather than solitary species. The gregariousness of prey species has been shown to reduce lion predation by reducing predator search efficiency (Fryxell et al. 2007) and improving group defense (Caro 2005), among other mechanisms. Third, the Serengeti‐Mara ecosystem is strongly seasonal and resource availability for grazers fluctuates dramatically over time (McNaughton 1979). Changes in resource availability may result in temporal variability in resource competition within and between species. Last, the Serengeti is one of the most well‐studied ecosystems with decades of prior research (Sinclair and Norton‐Griffiths 1979, Sinclair and Arcese 1995, Sinclair et al. 2008, 2015). Multiple years of camera trap data on the ungulate community (Swanson et al. 2015) along with long‐term predator monitoring (Packer et al. 2005, Packer 2019) and remotely sensed observations of resource availability offer an unprecedented opportunity to examine the drivers of mixed‐species groups over space and time.

**Fig. 1:**
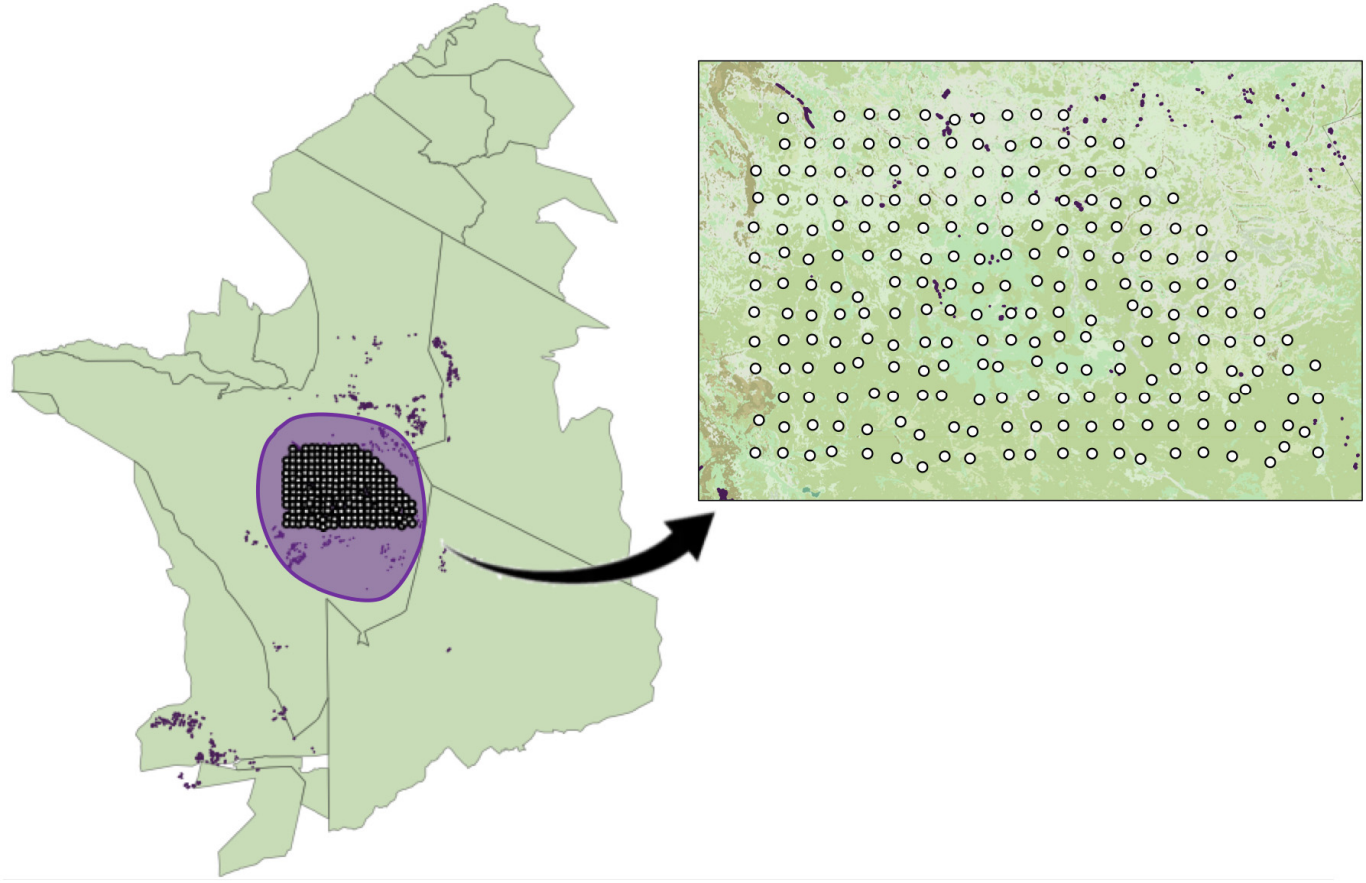
Study site. The map depicts the Serengeti‐Mara Ecosystem with the lion study area circled in purple, camera traps shown as white dots and kopjes shown in dark purple in both parts of the figure. The inset is a close‐up of the camera trap grid overlain on Landsat imagery of structural vegetation and kopjes.

Previous tests of the stress gradient hypothesis have focused predominately on changes in species interactions along a single stress gradient despite the prevalence of co‐occurring stresses in nature (He and Bertness 2014), such as the co‐occurring stresses of predation pressure and food stress. Here we test the stress gradient hypothesis using observations of mixed‐species groups among Serengeti grazers within the long‐term Serengeti Lion Project study area (Fig. 1) as a proxy for the potential for positive species interactions. We ask, specifically, does the probability of mixed‐species groups increase with increasing predation pressure or food stress?

We focus on six grazing ungulate species (Table 1) that comprise 85% of the diet of Serengeti lions both in terms of carcass numbers and kilograms of meat (Scheel and Packer 1995). Dietary overlap of the focal species in the Serengeti ranges from 24% to 74% (Hansen et al. 1985), which indicates the potential exists for resource competition between these species. As ambush hunters, Serengeti lions preferentially hunt where prey are easily catchable, such as in woodland habitat with denser woody vegetation or in viewsheds from rocky outcroppings called kopjes (Hopcraft et al. 2005). If mixed‐species groups enhance protection from predators, then mixed‐species groups will be more likely to occur as predation risk increases. If mixed‐species groups facilitate foraging, they will be more likely to occur as resource availability decreases. If, on the other hand, competition for resources limits mixed‐species groups, then mixed‐species groups will be more likely to occur as food availability increases.

## Materials and Methods

### Study site and species

Serengeti National Park, Tanzania (34°45'–35°14' E, 2°22'–2°55' S) is located within the Greater Serengeti‐Mara Ecosystem, an east African savannah dominated by the annual migration of 1.6 million wildebeest and zebra. This migration moves seasonally between Kenya and Tanzania, following a rainfall gradient from the wetter woodlands in the northwest to the southeastern short‐grass plains (Talbot and Talbot 1963, Sinclair and Norton‐Griffiths 1979).

We examined mixed‐species groups in six of the Serengeti’s most abundant grazing herbivores (Table 1): African buffalo (*Syncerus caffer*), Coke’s hartebeest (*Alcelaphus buselaphus cokii*), Thomson’s gazelle (*Eudorcas thomsonii*), topi (*Damaliscus lunatus jimela*), blue wildebeest (*Connochaetes taurinus*), and plains zebra (*Equus quagga*). All of these herbivores are gregarious, feed on similar resources (Gwynne and Bell 1968, Sinclair 1977), share the African lion as a common predator (Sinclair et al. 2003), and have been observed in mixed‐species groups (Anderson et al. 2016).

**Table 1:** Characteristics of study herbivores.

Common name	Scientific name	Mass (kg)	Residency	Proportion lion diet
African buffalo	*Syncerus caffer*	510–850 (m); 350–600 (f)	resident	13%
Blue wildebeest	*Connochaetes taurinus*	150–270 (m); 118–208 (f)	migratory	33%
Coke’s hartebeest	*Alcelaphus buselaphus cokii*	125–218 (m); 116–185 (f)	resident	2%
Plains zebra	*Equus quagga*	220–322 (m); 175–250 (f)	migratory	18%
Topi	*Damaliscus lunatus jimela*	(68–155)	resident	2%
Thomson's gazelle	*Eudorcas thomsonii*	20–25 (m); 16–21 (f)	migratory	17%

Notes

Mass is reported for males (m) and females (f). Proportion of lion diet was assessed by the number of carcasses.

*Sources:* Kingdon (1984), Scheel and Packer (1995), and Hopcraft et al. (2014).

A study of 10 Serengeti grazers reported the highest diet similarities among buffalo, Thompson’s gazelle, topi, and wildebeest (Hansen et al. 1985). The most common and most commonly consumed plant in the wet season by buffalo, Thomson’s gazelle, topi, wildebeest, and zebra was *Themeda trianda*; other plant genera consumed by these species included *Cynodon*, *Sporobolus*, *Brachiaria*, *Harpachne*, and *Aristida* (Hansen et al. 1985). In addition, the dry season diets of wildebeest and zebra contained the same plant genera. Despite dietary overlap and strong potential for competition between some grazers (e.g., topi and wildebeest; Murray and Illius 2000), specialization on different plant growth stages may facilitate coexistence among ecologically similar grazers. For example, Thomson’s gazelle, topi, and wildebeest utilize early and intermediate growth stages whereas buffalo and hartebeest utilize later growth stages (Murray and Brown 1993).

### Camera trap data

We deployed 210 camera traps in a 1,125‐km^2^ grid within the central part of Serengeti National Park. The camera trap grid lies within the Serengeti Lion Project’s long‐term (~50 yr) monitoring area (Packer et al. 2005, Packer 2019) and covers an intersection of open plains and woodland savannah (Fig. 1). Five camera traps were removed from the initial deployment due to access and maintenance and were therefore excluded from this study. Observations occurring between December and May were classified as wet season. Observations occurring between June and November were categorized as dry season.

Cameras were initially placed in 2010 and have operated continuously since 2011. We used ScoutGuard SG565 white‐flash trail cameras (trigger speed: 1.31 s; detection range: 25 m; Norcross, Georgia, USA). These cameras use passive infrared sensors triggered by heat and motion and operate continuously. Each camera was positioned within 250 m of the center of 5‐km^2^ grid cells on trees or metal poles and set approximately 50 cm above ground level to maximize capture of medium‐ to large‐sized vertebrates. Tall grass and hanging branches in front of each camera were trimmed every 6–8 weeks to provide an unobstructed field of view. All camera trap data in this study were collected between July 2010 and July 2015. An in‐depth description of the camera trap deployment is available elsewhere (Swanson et al. 2015).

Camera trap imagery was processed by volunteer members of the general public on the citizen science website, *Snapshot Serengeti*, who identified and counted the number of each species present in the photograph (photos *available online*).^6^ Multiple users (average: 27) scored each image, and the results were consolidated into a “consensus” classification based on agreement among users (Swanson et al. 2015). Prior validation of volunteer classifications against expert‐identified subset of the data demonstrated the consolidated classifications agreed with the “correct” classifications 97% of the time, and we improved this accuracy to 99% by limiting analyses to classifications with at least 60% agreement among volunteers (Swanson et al. 2016*b*). To prevent inflated counts of animals remaining in front of the camera traps for extended periods (e.g., triggering multiple images), consecutive captures from a camera within 10 minutes of each other containing the same species were consolidated into a single capture event (Palmer et al. 2017).

### Data analysis

#### Overview

In brief, we modeled the probability of mixed‐species groups occurring among the six focal species based on camera trap observations of mixed‐species groups and single‐species groups. We used a generalized linear mixed model where the response variable was the presence or absence of a mixed‐species group, which required a binomial distribution. The model included the following predictor variables: a continuous Normalized Difference Vegetational Index (NDVI) as a proxy for food availability, two continuous measures of predation risk (i.e., distance to kopjes, lion density), a categorical measure of predation risk (i.e., woodlands vs. plains habitat), a categorical variable for season (i.e., wet or dry) to account for differences in the number of migratory grazers, and a random effect for camera trap site to account for unexplained site‐level variation. Below we detail how the response variable and predictor variables were calculated.

#### Mixed‐species groups

We included observations of single‐species groups where more than one animal of a focal grazer species was photographed by a camera trap. We defined mixed‐species groups as a camera trap observation of a focal species with at least one individual of another focal species. All observations of mixed‐species groups contained two focal species. All observations within 30 m of permanent water sources were excluded to preclude analysis of species aggregating at water. Note that camera traps have a limited field of view, so photographic data likely underestimate the extent to which these species form mixed‐species groups, particularly those with three or more species. The unidirectional field of view may only record one species despite the existence of another species in the group occurring on another side of a camera. Such underestimates are likely exacerbated during the hours of darkness when the camera’s field of view is further restricted. We therefore tested for the latter effect by comparing results from the entire data set vs. photographs only collected during daylight hours, specifically between 06:00 and 19:00 local time.

To test whether mixed‐species groups differed from the number of associations expected based on the number of single‐species observations, we used a log‐likelihood ratio test (*G* test) (McDonald 2009). The null hypothesis of the *G* test is that the number of mixed‐species groups for a species is proportional to the number of observations of the species on its own. A significant result would indicate that observations of mixed‐species groups are not proportional to the number of observations per species.

#### Predictor variables

We used continuous NDVI as a proxy of food availability. Previous work has shown that this metric correlates with forage abundance and quality in our system (Anderson et al. 2010). We used NDVI data collected at a 250‐m resolution at 16‐d intervals (Tucker and Sellers 1986). NDVI measurements were extracted for each camera trap site and observation date and therefore varied over both space and time. We used an auto‐correlation function to test for temporal autocorrelation in the proportion of observations that were of mixed‐species groups per 16‐d NDVI sampling bin.

We approximated lion predation risk using three measures (1) distance to the nearest rocky outcropping (kopje) because kopjes provide enhanced viewsheds and are predictive of lion hunting success (Hopcraft et al. 2005), (2) lion density derived at a 1‐km^2^ scale, and (3) habitat type (plains or woodland) because Serengeti lions preferentially hunt in woodlands where habitat cover is greater (Hopcraft et al. 2005). Distances from each camera trap to the nearest kopje were derived from 1:50,000 digitized Aster images using QGIS v. 2.18.9 (Serengeti GIS and Data Center 2007). These distances varied spatially but were constant over time. To calculate lion encounter risk, we used lion monitoring data from the Serengeti Lion Project to construct pride territory boundaries for the 25 prides residing in the camera trap study area. Territory boundaries were defined as the 75% volume contour of kernel density estimates derived from VHF radio collar telemetry relocations; these values were weighted by both the number of lions in the pride and the duration of pride persistence in years between 2009–2014. The Serengeti Lion Project has traditionally used a 75% threshold (Mosser et al. 2009, Palmer et al. 2017) because of the potential sensitivity of kernel density estimates to sample size (Harris et al. 1990). Where territories overlapped, these values were summed at a 1‐km^2^ scale, and the final values were scaled based on the mean value of lion density across the entire study area. Independent estimates of lion density were calculated for wet and dry seasons, as territories tend to shift seasonally but are relatively stable across years. Thus, our continuous measurement of lion density varied spatially and between seasons, but did not vary among years. Habitat types were characterized for each camera trap location from 30‐m resolution vegetation layers (Serengeti GIS and Data Center 2007). Habitat type varied spatially but was constant over time.

We examined relationships between predictors of mixed‐species groups using the Wilcoxon rank sum test with continuity correction, which is a nonparametric test for assessing whether mean ranks differ and is appropriate for dependent samples from the same population. Specifically, we compared the 16‐d NDVI sampling values per camera trap for the dry season months versus the wet season months. Similarly, we compared the number of observations of grazers at each camera trap during the 16‐d NDVI sampling periods between dry season and wet season sampling periods. Because lion density estimates varied spatially between seasons but did not vary among years, we compared camera trap‐specific values of dry season lion density between plains and woodland habitats. We also compared camera trap‐specific values of wet season lion density between plains and woodland habitats. Last, we tested for differences in distance from camera traps to kopjes between plains and woodland habitats.

For the generalized linear mixed model, we scaled and centered continuous predictor variables; all coefficients reported herein are standardized beta coefficients and can be compared on a per unit basis. All analyses were performed in R version 3.5.1 (R Development Core Team 2019).

## Results

**Fig. 2:**
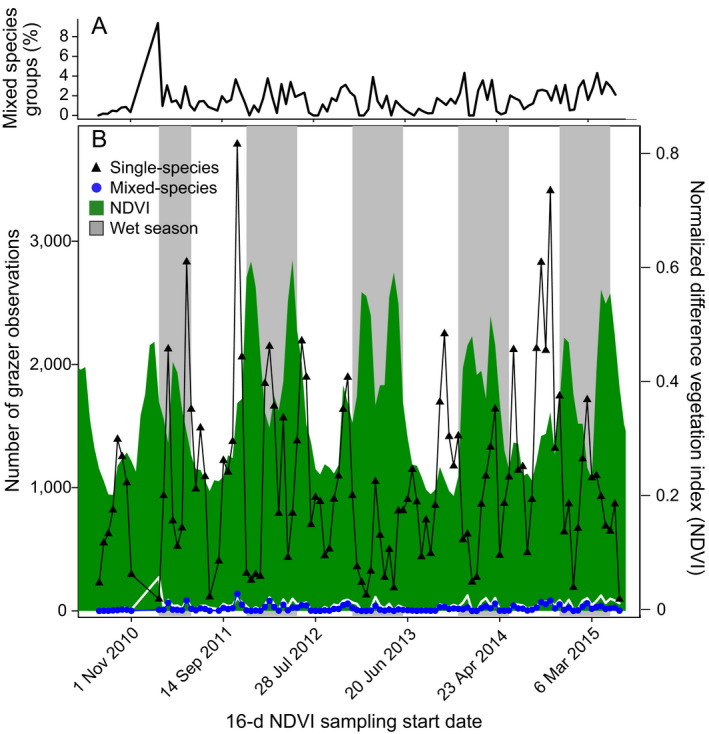
Temporal variation in Normalized Difference Vegetational Index (NDVI) and Serengeti grazer observations. (A) The percentage of observations of mixed‐species groups for each 16‐d NDVI sampling period is shown and (B) NDVI is shown in green shading, the number of single‐species observations are indicated by black triangles, and the number of mixed species observations are indicated by blue points. Gray shading represents the wet season. The white line illustrates the proportion of observations of mixed‐species groups, but note it is axis free.

We analyzed 115,136 unique camera trap observations of Serengeti grazers from 28,639 camera trap sampling days between July 2010 and July 2015. Of the total observations, 112,984 (98.1%) were observations of a single species. Zebra, Thomson’s gazelle, and wildebeest were the most commonly observed species with more than 27,000 single‐species observations each, buffalo and hartebeest had fewer than 6,500 single‐species observations each and topi were observed alone on 2,068 occasions (Table 2). Grazer observations fluctuated dramatically over time (Fig. 2), largely due to the movement of migratory zebra, Thomson’s gazelle, and wildebeest in and out of the study area (Appendix S1: Fig. S1). The auto‐correlation function showed a lack of significant temporal autocorrelation in the proportion of observations of mixed‐species groups per 16‐d NDVI sampling bin (Appendix S1: Fig. S2).

**Table 2:** The number of observations of each focal species individually, in mixed‐species groups, and for each species pair.

Common name	No. observations			No. mixed‐species groups for each pair of species				
	Single species	Mixed species	Mixed species observations (%)	Buffalo	Thomson's gazelle	Hartebeest	Topi	Wildebeest
Buffalo	6,397	19	0.30					
Thomson's gazelle	32,176	222	0.69	6				
Hartebeest	5,754	74	1.29	4	53			
Topi	2,068	25	1.21	2	20	0		
Wildebeest	27,941	834	2.98	4	90	12	5	
Zebra	38,648	978	2.53	22	275	79	23	1,557

Notes

The number of observations of mixed‐species groups for each species on the left side of the table is one‐half of the sum for that species for its number of mixed‐species groups on the right side of the table. The right side of the table counts each mixed‐species group twice because it counts a mixed‐species group from the perspective of each species.

Camera traps recorded 2,152 occurrences of mixed‐species groups. The number of mixed‐species groups varied among species pairs (Table 2) and over time (Fig. 2). Overall, 91% of all mixed‐species groups contained zebra. The most commonly observed mixed‐species groups were of zebra and wildebeest (*N* = 1,557 observations) while at the other extreme, topi and hartebeest were never observed together (Table 2). Log‐likelihood ratio (*G* tests) of independence showed that the number of mixed‐species observations differed significantly from the proportion expected based on the number of single‐species observations for all six species (*G* = 691.41, df = 5 *P* < 0.001), for the three migratory species (*G* = 527.31, df = 2, *P* < 0.001), and for the three resident species (*G* = 44.27, df = 2, *P* < 0.001), which suggests that the mixed‐species groups did not occur based on chance alone.

We examined multiple predictors of mixed‐species groups and here we describe relationships between the predictors we examined (Appendix S1: Fig. S3). Of the 108 16‐d NDVI sampling periods, 52 sampling periods were during the dry season and 56 were during the wet season. Of the 205 camera trap sampling locations, 145 camera traps were located in plains habitat and 60 camera traps were located in woodland habitat. There were significantly more observations of grazers during dry season sampling periods than wet season sampling periods due to seasonal migration (Wilcoxon rank sum test: *W* = 1,924, *P* = 0.004) and NDVI of the study area was significantly higher during the wet season sampling periods (Wilcoxon rank sum test: *W* = 18,410,590, *p* < 0.001). Lion density was not significantly higher in plains habitat than in woodland habitat during the wet season sampling periods when migration occurred (Wilcoxon rank sum test: *W* = 4,296, *P* = 0.890), and did not differ significantly between plains and woodland habitats during the dry season sampling periods (Wilcoxon rank sum test: *W* = 4,352.5, *P* = 0.996). Distance from camera traps to the nearest kopje did not differ significantly between woodland and plains habitats (Wilcoxon rank sum test: *W* = 3,630, *P* = 0.063).

**Fig. 3:**
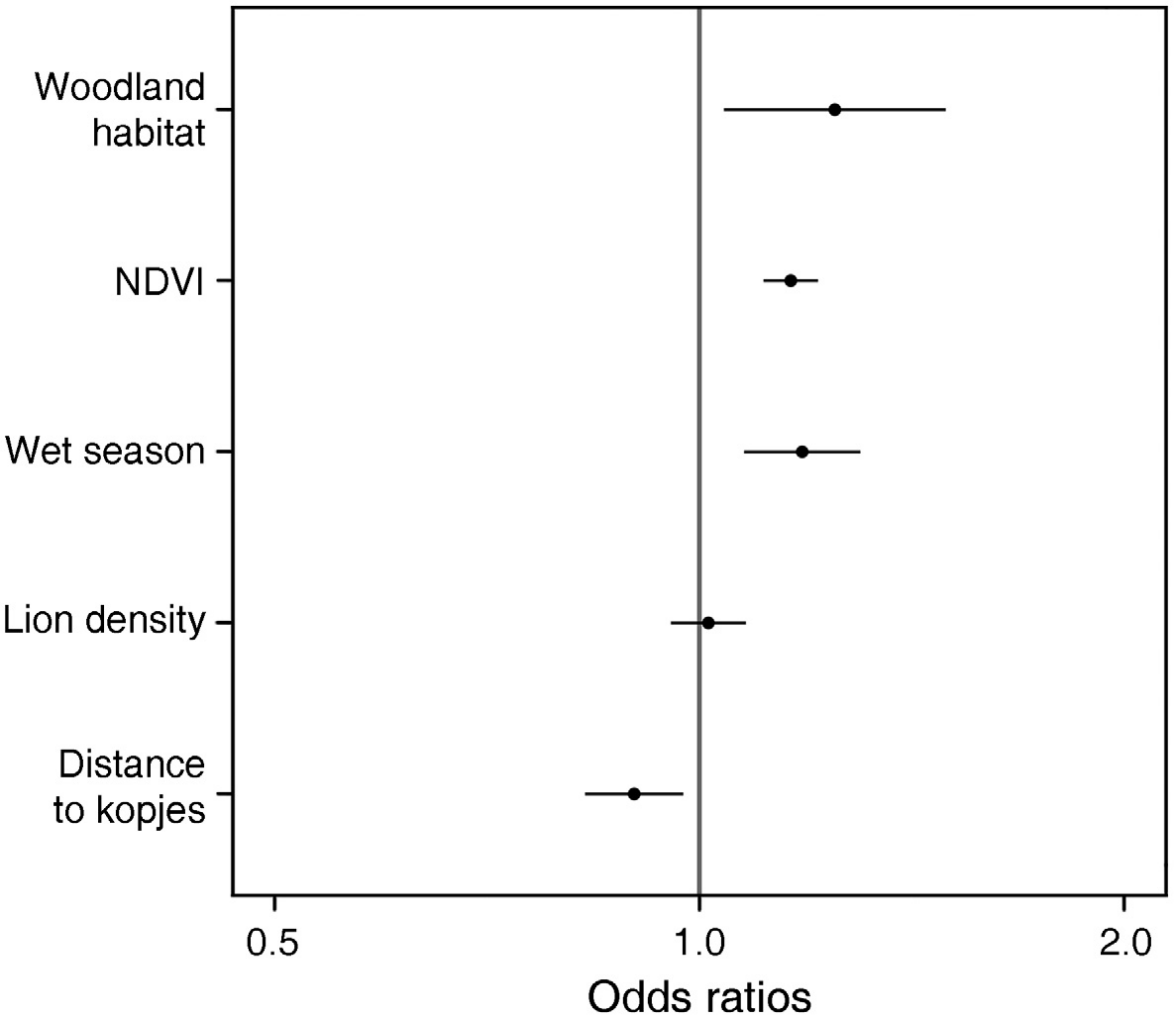
Model results for predictors of mixed‐species groups. The probability of a mixed‐species group occurring was significantly higher in woodland habitat, when NDVI was high, and during the wet season. The probability of a mixed‐species group occurring increased as distance to kopjes declined (i.e., mixed‐species groups occurred more often closer to rocky viewsheds). The plot of standardized coefficients depicts the fixed effect terms from the generalized linear mixed model predicting the occurrence of mixed‐species groups. Points indicate estimates and lines indicate standard errors. Odds ratios > 1 are positive effects whereas odds ratios < 1 are negative effects. An odds ratio estimate with standard errors overlapping one indicates a lack of statistical significance when alpha = 0.05.

We used a generalized linear mixed‐effects model to test the effects of predation risk and food availability on the probability of mixed‐species groups in Serengeti grazers (Fig. 3). Mixed‐species groups were 1.25 times more likely to occur in woodland habitats than in the plains (estimate = 0.221, SE = 0.09, *P* = 0.016) and 1.11 times less likely to occur for each unit increase in distance from kopjes (estimate = −0.106, SE = 0.04, *P* = 0.009). They were 1.16 times more likely to occur for each unit increase in NDVI (estimate = 0.149, SE = 0.023, *P* < 0.001) and 1.18 times more likely to occur during the wet season (estimate = 0.168, SE = 0.048, *P* < 0.001). Shorter camera trap detection distances in woodlands compared to plains could result in underestimates of mixed‐species groups in woodland habitat. However, the model estimate indicating that mixed‐species groups were significantly more likely to occur in woodland than plains habitat suggests that these results are robust to such a detection bias.

Because kopjes are not distributed randomly throughout the camera trap sampling area and the increased sighting distance that kopjes provide to lions is limited, we repeated the generalized linear mixed‐effects model using the subset of observations within 1,000 m from a kopje. This subset of data included 22,784 total observations from 30 camera traps with 438 observations of mixed‐species groups. Consistent with the complete data set, mixed‐species groups were 1.28 times less likely to occur for each unit increase in distance from kopjes (estimate = −0.246, SE = 0.112, *P* = 0.023) and were 1.13 times more likely to occur for each unit increase in NDVI (estimate = 0.125, SE = 0.051, *P* = 0.015).

The results of all analyses were robust to the exclusion of nocturnal observations (Appendix S1: Table S1, Figs. S4, S5).

## Discussion

We tested the stress gradient hypothesis using the occurrence of mixed‐species groups as a proxy for the potential for positive species interactions and modeling whether predation risk and food availability predicted the likelihood of mixed‐species groups in Serengeti grazers. Mixed‐species groups among the six species of ungulates were uncommon overall (1.9% of observations), but occurred in proportions differently than were expected based on the number of single‐species observations. Notably, lion density did not significantly predict mixed‐species groups. Instead, mixed‐species groups were more likely to occur in risky areas, specifically in woodland habitat and closer to rocky outcroppings that provide lions with viewsheds for scoping prey. Previous research has shown that Serengeti lions preferentially hunt in woodland habitat where vegetative cover is greater (Hopcraft et al. 2005). In addition, herbivores avoid areas with dense trees, such as woodlands, where visibility is lower (Riginos and Grace 2008). The higher probabilities of mixed‐species groups in woodland habitat and near rocky outcroppings are consistent with the prediction that mixed‐species groups are more likely to occur as predation pressure increases. Our finding that lion density did not affect grouping behavior yet woodland habitat and proximity to kopjes did predict mixed‐species groups suggests that these landscape characteristics may influence grazer perceptions of risk, and more specifically suggests that these landscape characteristics may influence migratory zebra perceptions of risk given that they participated in the vast majority of mixed‐species observations.

Mixed‐species groups were significantly less likely to occur when NDVI was low, which is consistent with the interpretation that resource competition limits mixed‐species groups. Notably, Serengeti wildebeest and zebra have nearly complete dietary overlap during the dry season during which NDVI is lower than the wet season (Hansen et al. 1985) and the most commonly observed mixed‐species group was of wildebeest and zebra. Our results are therefore consistent with support for the stress gradient hypothesis in animals along a consumer pressure gradient but not along a food stress gradient. Instead, our results highlight a potential co‐occurring stress from resource availability on the formation of mixed species groups, which is consistent with the body of work addressing trade‐offs between foraging and risk in savanna ungulates (Hopcraft et al. 2010).

The stress gradient hypothesis has only recently been applied to animal communities and has mostly been tested experimentally in invertebrates; these few studies have found some support that positive interactions increase along resource stress gradients (Fugere et al. 2012, Dangles et al. 2013, 2018). An aquatic field study found that positive interactions were more important for reproductive success as physical stress increased (Peoples et al. 2015), but tests of positive interactions resulting from high consumer pressure have been lacking. Our study therefore provides one of the first tests of whether positive interactions between animal species may increase under conditions of high consumer pressure.

Previous studies have documented the importance of predation in structuring the Serengeti ungulate community (Sinclair 1985, Sinclair et al. 2003, Hopcraft et al. 2012). Carnivore diets within the Serengeti are nested based on the body sizes of both predators and prey. Smaller‐bodied ungulates are consumed by more species of predators than are larger‐bodied ungulate species, but each carnivore species prefers a narrow range of prey sizes within their diet range (Sinclair et al. 2003). After accounting for the higher probability of mixed‐species groups during the migration (i.e., wet season), mixed‐species groups were still significantly more likely to occur in risky woodland habitats and were more often observed near rocky outcroppings that predators use as vantage points. These results are consistent with the interpretation that mixed‐species groups are more likely to form when predation risk is high. The most commonly observed mixed‐species groups in this study consisted of zebra and wildebeest. Zebra have been shown to use wildebeest for protection from predators in the Serengeti (Sinclair 1985) and in other savanna–woodland ecosystems (de Boer and Prins 1990, Thaker et al. 2010, Kiffner et al. 2014). Migrating species, such as wildebeest and zebra, continuously move through unfamiliar areas whereas resident species, such as topi and hartebeest, that reside within familiar areas may rely on different cues to reduce their risks of predation. Schmitt et al. (2014) demonstrated that predator detection benefits are significantly increased in mixed‐species groups of zebra and wildebeest compared to single species zebra groups. Furthermore, zebra in mixed‐species group have lower vigilance levels and therefore can spend more time feeding than zebra in single species groups (Stears et al. 2020). Such benefits obtained through mixed‐species groups likely explain why zebra often form mixed‐species groups. Our results further elucidate the risky spatial conditions under which mixed‐species groups are more likely to occur and suggest that these physical landscape characteristics cue a landscape of fear that results in spatial variation in anti‐predator behavior (Gaynor et al. 2019).

Our results are also consistent with the alternative hypothesis that resource competition limits mixed‐species groups of Serengeti grazers because mixed‐species groups were less likely to occur when forage availability was low. State‐space modeling of zebra movement has previously shown that zebra compromise safety when resources are scarce (Hopcraft et al. 2014). Zebra may therefore refrain from forming mixed‐species groups with wildebeest when resources are scarce. Furthermore, topi, hartebeest, and buffalo have been shown to compete for similar resources (Murray and Brown 1993, Murray and Illius 2000). The low number of mixed‐species groups among topi, hartebeest, and buffalo is consistent with the interpretation that mixed‐species groups are limited by resource competition and likely due to the potential costs of competition outweighing the potential anti‐predator benefits of mixed‐species herding.

While we found that mixed‐species group formation did not appear to provide immediate benefits for participating species in terms of resource facilitation, there is strong evidence that facilitation does occur among grazers in a time‐delayed manner. For example, migratory wildebeest facilitate Thomson’s gazelle by grazing heavily, which prepares plant communities for consumption by Thomson’s gazelle in the following dry season (McNaughton 1976). Protein content and digestibility are often inversely related to grass maturity, thus, younger stems provide higher quality food for grazers (van Soest 1982, Wilmshurst et al. 1999). Stem regrowth following wildebeest grazing provides higher nutritional content for Thomson’s gazelles. Lastly, facilitation among savanna grazers also occurs when megaherbivores, such as buffalo, maintain short grass swards that feed smaller herbivores (Cromsigt and Olff 2008).

We focused on six ungulate species that have similar herbivorous diets and comprise 85% of the largest predator’s diet, but the Serengeti supports a hyper‐diverse large mammal community with 10 large carnivore species and at least 28 ungulate species (Sinclair and Norton‐Griffiths 1979). Notably, there is substantial dietary overlap between lions and spotted hyena (*Crocuta crocuta*; Cooper et al. 1999, Honer et al. 2002). In addition, Thomson’s gazelles are a preferred food of cheetahs (*Acinonyx jubates*; Hayward et al. 2006). While we were unable to directly test the effects of spotted hyena or cheetah density on the occurrence of mixed‐species groups due to lack of comparable home range data, space use by spotted hyenas and cheetahs is positively associated with lions in the Serengeti and elsewhere (Broekhuis et al. 2013, Swanson et al. 2016*a*). Overlapping habitat use among lions, spotted hyenas, and cheetahs suggests similar spatial patterns of predation risk from multiple predators. The significantly higher occurrence of mixed‐species groups in woodland habitat and near kopjes may reduce predation risk from multiple co‐occurring predators. Last, the six ungulate species we examined may benefit from mixed‐species groups with other herbivores not considered in this study. For example, Thomson’s gazelle and Grant’s gazelle form mixed‐species groups that provide antipredator benefits from cheetah (Fitzgibbon 1990).

Few empirical studies of the stress gradient hypothesis in any taxa have examined the roles of multiple co‐occurring stressors across a single habitat (He and Bertness 2014). We have used the occurrence of mixed‐species groups as a proxy for positive species interactions and have shown that mixed‐species groups of grazers occur at frequencies differently than expected based on the number of single‐species observations, vary significantly over space and time, and are more likely to occur in spatially risky areas where prey visibility is low (i.e., in woodland habitat) and predator visibility is high (i.e., near rocky outcroppings). Our results are consistent with the interpretation that increased resource competition may limit the occurrence of mixed‐species groups when resource availability is low. Despite the relatively widespread existence of mixed‐species groups within primates, cetaceans and ungulates, obtaining sufficient observational data for rigorous statistical analysis of the drivers of mixed‐species groups in mammals has hitherto precluded quantitative analysis as a function of ecological context (Cords and Wursig 2014). Camera trap data used in this study likely underestimate the occurrence of mixed‐species groups due to the cameras’ limited view, nonetheless the Serengeti‐Mara ecosystem has provided a valuable opportunity to evaluate predictions of the stress gradient hypothesis under co‐occurring stressors. Our results are consistent with support for the stress gradient hypothesis along a consumer pressure gradient for mobile animals thus providing a novel link between extensive research in plant community ecology on the stress gradient hypothesis and research in animal ecology on trade‐offs between foraging and risk.

## Supporting information

Appendix S1

## Data Availability

Code and files necessary to replicate results and figures are available on Zenodo: http://doi.org/10.5281/zenodo.3922940

## Footnotes

^6^
www.snapshotserengeti.org

## References

[ecy3163-bib-0001] Anderson, T. M. , J. G. Hopcraft , S. Eby , M. Ritchie , J. B. Grace , and H. Olff . 2010 Landscape‐scale analyses suggest both nutrient and antipredator advantages to Serengeti herbivore hotspots. Ecology 99:1519–1529. 10.1890/09-0739.1 20503883

[ecy3163-bib-0002] Anderson, T. M. , S. White , B. Davis , R. Erhardt , M. Palmer , A. Swanson , M. Kosmala , and C. Packer . 2016 The spatial distribution of African savannah herbivores: species associations and habitat occupancy in a landscape context. Philosophical Transactions of the Royal Society B 371:20150314. 10.1098/rstb.2015.0314 PMC4978872 27502379

[ecy3163-bib-0003] Barrio, I. C. , D. S. Hik , C. G. Bueno , and J. F. Cahill . 2012 Extending the stress‐gradient hypothesis—is competition among animals less common in harsh environments? Oikos 122:516–523.

[ecy3163-bib-0004] Beauchamp, G. , and G. D. Ruxton . 2005 Harvesting resources in groups or alone: the case of renewing patches. Behavioral Ecology 16:989–993.

[ecy3163-bib-0005] Bertness, M. D. , and R. Callaway . 1994 Positive interactions in communities. Trends in Ecology & Evolution 9:191–193. 21236818 10.1016/0169-5347(94)90088-4

[ecy3163-bib-0006] Broekhuis, F. , G. Cozzi , M. Valeix , J. W. McNutt , and D. W. Macdonald . 2013 Risk avoidance in sympatric large carnivores: reactive or predictive? Journal of Animal Ecology 82:1097–1105. 10.1111/1365-2656.12077 23692142

[ecy3163-bib-0007] Bruno, J. F. , J. J. Stachowicz , and M. D. Bertness . 2003 Inclusion of facilitation into ecological theory. Trends in Ecology & Evolution 18:119–125.

[ecy3163-bib-0008] Caro, T. 2005 Antipredator defenses in birds and mammals. The University of Chicago Press, Chicago, Illinois, USA.

[ecy3163-bib-0009] Cooper, S. M. , K. E. Holekamp , and L. Smale . 1999 A seasonal feast: long‐term analysis of feeding behaviour in the spotted hyaena (*Crocuta crocuta*). African Journal of Ecology 37:149–160.

[ecy3163-bib-0010] Cords, M. , and B. Wursig . 2014 A mix of species: associations of heterospecifics among primates and dolphins Pages 409–431 *in* Yamagiwa J., and Karczmarski L., editors. Primates and Cetaceans: field research and conservation of complex mammalian societies. Springer, Tokyo, Japan.

[ecy3163-bib-0011] Cromsigt, J. P. G. M. , and H. Olff . 2008 Dynamics of grazing lawn formation: an experimental test of the role of scale‐dependent processes. Oikos 117:1444–1452.

[ecy3163-bib-0012] Dangles, O. , M. Herrera , and F. Anthelme . 2013 Experimental support of the stress‐gradient hypothesis in herbivore‐herbivore interactions. New Phytologist 197:405–408. 23174037 10.1111/nph.12080

[ecy3163-bib-0013] Dangles, O. , M. Herrera , C. Carpio , and C. J. Lortie . 2018 Facilitation costs and benefits function simultaneously on stress gradients for animals. Proceedings of the Royal Society B: Biological Sciences 285:20180983 10.1098/rspb.2018.0983 PMC6125904 30135157

[ecy3163-bib-0014] de Boer, W. F. , and H. H. T. Prins . 1990 Large herbivores that strive mightily but eat and drink as friends. Oecologia 82:264–274. 28312674 10.1007/BF00323544

[ecy3163-bib-0015] Diamond, J. M. 1975 Assembly of species communities Pages 342–444 *in* Cody M. L. and Diamond J. M., editors. Ecology and evolution of communities. Harvard University Press, Cambridge, UK.

[ecy3163-bib-0016] Elton, C. S. 1946 Competition and the structure of ecological communities. Journal of Animal Ecology 15:54–68.

[ecy3163-bib-0017] Fitzgibbon, C. D. 1990 Mixed‐species grouping in Thomson’s and Grant’s gazelles: the antipredator benefits. Animal Behaviour 39:1116–1126.

[ecy3163-bib-0018] Foster, W. A. , and J. E. Treherne . 1981 Evidence for the dilution effect in the selfish herd from fish predation on a marine insect. Nature 293:466–467.

[ecy3163-bib-0019] Fryxell, J. M. 1991 Forage quality and aggregation by large herbivores. American Naturalist 138:478–498.

[ecy3163-bib-0020] Fryxell, J. M. , A. Mosser , A. R. E. Sinclair , and C. Packer . 2007 Group formation stabilizes predator‐prey dynamics. Nature 449:1041–1044. 17960242 10.1038/nature06177

[ecy3163-bib-0021] Fryxell, J. M. , J. Wilmshurst , A. R. E. Sinclair , D. T. Haydon , R. D. Holt , and P. A. Abrams . 2005 Landscape scale, heterogeneity, and the viability of Serengeti grazers. Ecology Letters 8:328–335.

[ecy3163-bib-0022] Fugere, V. , P. Andino , R. Espinosa , F. Anthelme , D. Jacobsen , and O. Dangles . 2012 Testing the stress‐gradient hypothesis with aquatic detritivorous invertebrates: insights for biodiversity‐ecosystem functioning research. Journal of Animal Ecology 81:1259–1267. 22548624 10.1111/j.1365-2656.2012.01994.x

[ecy3163-bib-0023] Gaynor, K. M. , J. S. Brown , A. D. Middleton , M. E. Power , and J. S. Brashares . 2019 Landscapes of fear: spatial patterns of risk perception and response. Trends in Ecology & Evolution 34:355–368. 30745252 10.1016/j.tree.2019.01.004

[ecy3163-bib-0024] Gwynne, M. D. , and R. H. V. Bell . 1968 Selection of grazing components by grazing ungulates in the Serengeti National Park. Nature 220:390–393. 5684885 10.1038/220390a0

[ecy3163-bib-0025] Hansen, R. M. , M. M. Mugambi , and S. M. Bauni . 1985 Diets and trophic ranking of ungulates of the northern Serengeti. Journal of Wildlife Management 49:823–829.

[ecy3163-bib-0026] Harris, S. , W. J. Cresswell , P. G. Forde , W. J. Trewhella , T. Woollard , and S. Wray . 1990 Home‐range analysis using radio‐tracking data ‐ a review of problems and techniques particularly as applied to the study of mammals. Mammal Review 20:97–123.

[ecy3163-bib-0027] Hayward, M. W. , M. Hofmeyr , J. O'Brien , and G. I. H. Kerley . 2006 Prey preferences of the cheetah (*Acinonyx jubatus*) (Felidae : Carnivora): morphological limitations or the need to capture rapidly consumable prey before kleptoparasites arrive? Journal of Zoology 270:615–627.

[ecy3163-bib-0028] He, Q. , and M. D. Bertness . 2014 Extreme stresses, niches, and positive species interactions along stress gradients. Ecology 95:1437–1443. 25039207 10.1890/13-2226.1

[ecy3163-bib-0029] He, Q. , M. D. Bertness , and A. H. Altieri . 2013 Global shifts towards positive species interactions with increasing environmental stress. Ecology Letters 16:695–706. 23363430 10.1111/ele.12080

[ecy3163-bib-0030] Heymann, E. W. , and S. S. Hsia . 2015 Unlike fellows ‐ a review of primate‐non‐primate associations. Biological Reviews 90:142–156. 24661546 10.1111/brv.12101

[ecy3163-bib-0031] Honer, O. P. , B. Wachter , M. L. East , and H. Hofer . 2002 The response of spotted hyaenas to long‐term changes in prey populations: functional response and interspecific kleptoparasitism. Journal of Animal Ecology 71:236–246.

[ecy3163-bib-0032] Hopcraft, J. G. , T. M. Anderson , S. Perez‐Vila , E. Mayemba , and H. Olff . 2012 Body size and the division of niche space: food and predation differentially shape the distribution of Serengeti grazers. Journal of Animal Ecology 81:201–213. 21801174 10.1111/j.1365-2656.2011.01885.x

[ecy3163-bib-0033] Hopcraft, J. G. C. , J. M. Morales , H. L. Beyer , M. Borner , E. Mwangomo , A. R. E. Sinclair , H. Olff , and D. T. Haydon . 2014 Competition, predation, and migration: individual choice patterns of Serengeti migrants captured by hierarchical models. Ecological Monographs 84:355–372.

[ecy3163-bib-0034] Hopcraft, J. G. C. , H. Olff , and A. R. E. Sinclair . 2010 Herbivores, resources and risks: alternating regulation along primary environmental gradients in savannas. Trends in Ecology & Evolution 25:119–128. 19767121 10.1016/j.tree.2009.08.001

[ecy3163-bib-0035] Hopcraft, J. G. , A. R. E. Sinclair , and C. Packer . 2005 Planning for success: Serengeti lions seek prey accessibility rather than abundance. Journal of Animal Ecology 74:559–566.

[ecy3163-bib-0036] Kamilar, J. M. , and L. Beaudrot . 2013 Understanding primate communities: recent developments and future directions. Evolutionary Anthropology 22:174–185. 23943271 10.1002/evan.21361

[ecy3163-bib-0037] Kiffner, C. , J. Kioko , C. Leweri , and S. Krause . 2014 Seasonal patterns of mixed species groups in large east African mammals. PLoS ONE 9:e113446. 25470495 10.1371/journal.pone.0113446 PMC4254287

[ecy3163-bib-0038] Kingdon, J. 1984 East African mammals. University of Chicago Press, Chicago, Illinois, USA.

[ecy3163-bib-0039] Krebs, J. R. 1973 Social‐lLearning and significance of mixed‐species flocks of Chickadees (*Parus* spp). Canadian Journal of Zoology 51:1275–1288.

[ecy3163-bib-0040] McDonald, J. H. 2009 Handbook of biological statistics. Sparky House Publishing, Baltimore, Maryland, USA.

[ecy3163-bib-0041] McNaughton, S. J. 1976 Serengeti migratory wildebeest: facilitation of energy flow by grazing. Science 191:92–94. 17834943 10.1126/science.191.4222.92

[ecy3163-bib-0042] McNaughton, S. J. 1979 Grassland‐herbivore dynamics Pages 46–81 *in* Sinclair A. R. E. and Norton‐Griffiths M., editors. Serengeti: dynamics of an ecosystem. University of Chicago Press, Chicago, Illinois. USA.

[ecy3163-bib-0043] Miller, R. C. 1922 The significance of the gregarious habit. Ecology 3:122–126.

[ecy3163-bib-0044] Mosser, A. , J. M. Fryxell , L. Eberly , and C. Packer . 2009 Serengeti real estate: density vs. fitness‐based indicators of lion habitat quality. Ecology Letters 12:1050–1060. 19708970 10.1111/j.1461-0248.2009.01359.x

[ecy3163-bib-0045] Murray, M. G. , and D. Brown . 1993 Niche separation of grazing ungulates in the Serengeti: an experimental test. Journal of Animal Ecology 62:380–389.

[ecy3163-bib-0046] Murray, M. G. , and A. W. Illius . 2000 Vegetation modification and resource competition in grazing ungulates. Oikos 89:501–508.

[ecy3163-bib-0047] Packer, C. 2019 The African lion: a long history of interdisciplinary research. Frontiers in Ecology and Evolution 7:1–6.

[ecy3163-bib-0048] Packer, C. , R. Hilborn , A. Mosser , B. Kissui , M. Borner , J. G. Hopcraft , J. Wilmshurst , S. A. R. Mduma , and A. R. E. Sinclair . 2005 Ecological change, group territoriality, and population dynamics in Serengeti lions. Science 307:390–393. 15662005 10.1126/science.1105122

[ecy3163-bib-0049] Palmer, M. S. , J. Fieberg , A. K. Swanson , M. Kosmala , and C. Packer . 2017 A ‘dynamic’ landscape of fear: prey responses to spatiotemporal variations inpredation risk across the lunar cycle. Ecology Letters 20:1364–1373. 28901034 10.1111/ele.12832

[ecy3163-bib-0081] Peoples, B. K. , L. A. Blanc , and E. A. Frimpong . 2015 Lotic cyprinid communities can be structured as nest webs and predicted by the stress‐gradient hypothesis. Journal of Animal Ecology 84:1666–1677. 26250466 10.1111/1365-2656.12428

[ecy3163-bib-0050] Pulliam, H. R. 1973 On the advantages of flocking. Journal of Theoretical Biology 38:419–422. 4734745 10.1016/0022-5193(73)90184-7

[ecy3163-bib-0051] R Development Core Team . 2019 R: a language and environment for statistical computing. R Foundation for Statistical Computing, Vienna, Austria. www.R‐project.org

[ecy3163-bib-0052] Riginos, C. , and J. B. Grace . 2008 Savanna tree density, herbivores, and the herbaceous community: bottom‐up vs. top‐down effects. Ecology 89:2228–2238. 18724733 10.1890/07-1250.1

[ecy3163-bib-0053] Scheel, D. , and C. Packer . 1995 Variation in predation by lions: tracking a movable feast Pages 299–314 *in* Sinclair A. R. E. and Arcese P., editors. Serengeti II: dynamics, management and conservation of an ecosystem. University of Chicago, Chicago, Illinois, USA.

[ecy3163-bib-0054] Schmitt, M. H. , K. Stears , and A. M. Shrader . 2016 Zebra reduce predation risk in mixed‐species herds by evesdropping on cues from giraffe. Behavioral Ecology 27:1073–1077.

[ecy3163-bib-0055] Schmitt, M. H. , K. Stears , C. C. Wilmers , and A. M. Shrader . 2014 Determining the relative importance of dilution and detection for zebra foraging in mixed‐species herds. Animal Behaviour 96:151–158.

[ecy3163-bib-0056] Schoener, T. W. 1971 Theory of feeding strategies. Annual Review of Ecology and Systematics 2:369–404.

[ecy3163-bib-0057] Serengeti GIS & Data Centre . 2007 Serengeti vegetation map [shape‐ files and metadata]. https://www.serengetidata.org

[ecy3163-bib-0058] Shaw, E. 1962 The schooling of fishes. Scientific American 206:128–141.14458553

[ecy3163-bib-0059] Sinclair, A. R. E. 1977 The African buffalo. University of Chicago Press, Chicago, Illinois, USA.

[ecy3163-bib-0060] Sinclair, A. R. E. 1985 Does interspecific competition or predation shape the African ungulate community. Journal of Animal Ecology 54:899–918.

[ecy3163-bib-0061] Sinclair, A. R. E. , and P. Arcese . 1995 Serengeti II: dynamics, management, and conservation of an ecosystem. University of Chicago Press, Chicago, Illinois, USA.

[ecy3163-bib-0062] Sinclair, A. R. E. , S. A. R. Mduma , and J. S. Brashares . 2003 Patterns of predation in a diverse predator‐prey system. Nature 425:288–290. 13679915 10.1038/nature01934

[ecy3163-bib-0063] Sinclair, A. R. E. , K. L. Metzger , S. A. R. Mduma , and J. M. Fryxell . 2015 Serengeti IV: Sustaining biodiversity in a coupled human‐natural system. University of Chicago Press, Chicago, Illinois, USA.

[ecy3163-bib-0064] Sinclair, A. R. E. , and M. Norton‐Griffiths . 1979 Serengeti: dynamics of an ecosystem. University of Chicago Press, Chicago, Illinois, USA.

[ecy3163-bib-0065] Sinclair, A. R. E. , C. Packer , S. A. R. Mduma , and J. M. Fryxell . 2008 Serengeti III: human impacts on ecosystem dynamics. University of Chicago Press, Chicago, Illinois, USA.

[ecy3163-bib-0066] Sridhar, H. , G. Beauchamp , and K. Shanker . 2009 Why do birds participate in mixed‐species foraging flocks? A large‐scale synthesis. Animal Behaviour 78:337–347.

[ecy3163-bib-0067] Stachowicz, J. J. 2001 Mutualism, facilitation, and the structure of ecological communities. BioScience 51:235–246.

[ecy3163-bib-0068] Stears, K. , M. H. Schmitt , C. C. Wilmers , and A. M. Shrader . 2020 Mixed‐species herding levels the landscape of fear. Proceedings of the Royal Society B 287:20192555. 32126952 10.1098/rspb.2019.2555 PMC7126070

[ecy3163-bib-0069] Stensland, E. , A. Angerbjorn , and P. Berggren . 2003 Mixed species groups in mammals. Mammal Review 33:205–223.

[ecy3163-bib-0070] Swanson, A. , T. Arnold , M. Kosmala , J. Forester , and C. Packer . 2016a In the absence of a "landscape of fear": How lions, hyenas, and cheetahs coexist. Ecology and Evolution 6:8534–8545. 28031805 10.1002/ece3.2569 PMC5167031

[ecy3163-bib-0071] Swanson, A. , M. Kosmala , C. Lintott , and C. Packer . 2016b A generalized approach for producing, quantifying, and validating citizen science data from wildlife images. Conservation Biology 30:520–531. 27111678 10.1111/cobi.12695 PMC4999033

[ecy3163-bib-0072] Swanson, A. , M. Kosmala , C. Lintott , R. Simpson , A. T. Smith , and C. Packer . 2015 Snapshot Serengeti, high‐frequency annotated camera trap images of 40 mammalian species in an African savanna. Scientific Data 2:150026. 26097743 10.1038/sdata.2015.26 PMC4460915

[ecy3163-bib-0073] Talbot, L. M. , and M. H. Talbot . 1963 The wildebeest in western Maasailand, E. Africa. Wildlife Society, Seattle, Washington, USA.

[ecy3163-bib-0074] Terborgh, J. 1990 Mixed flocks and polyspecific associations—costs and benefits of mixed groups to birds and monkeys. American Journal of Primatology 21:87–100. 31963979 10.1002/ajp.1350210203

[ecy3163-bib-0075] Thaker, M. , A. T. Vanak , C. R. Owen , M. B. Ogden , and R. Slotow . 2010 Group dynamics of zebra and wildebeest in a woodland savanna: Effects of predation risk and habitat density. PLoS ONE 5:e12758. 20862216 10.1371/journal.pone.0012758 PMC2942830

[ecy3163-bib-0076] Thompson, J. N. 1999 Specific hypotheses on the geographic mosaic of coevolution. American Naturalist 153:S1–S14.

[ecy3163-bib-0077] Tucker, C. J. , and P. J. Sellers . 1986 Satellite remote sensing for primary production. International Journal of Remote Sensing 7:1395–1416.

[ecy3163-bib-0078] van Ommeren, R. J. , and T. G. Whitham . 2002 Changes in interactions between juniper and mistletoe mediated by shared avian frugivores: parasitism to potential mutualism. Oecologia 130:281–288. 28547152 10.1007/s004420100792

[ecy3163-bib-0079] van Soest, P. J. 1982 Nutritional ecology of the ruminant. O&B Books, Corvallis, Oregon, USA.

[ecy3163-bib-0080] Wilmshurst, J. , J. M. Fryxell , and P. Colucci . 1999 What constrains daily intake in Thomson’s gazelles? Ecology 80:2338–2347.

