# Supplementary material for: Mixed‐species groups of Serengeti grazers: a test of the stress gradient hypothesis
Source: Ecology. 2020 Sep 9;101(11):e03163. doi: 10.1002/ecy.3163 (PMC7685109; doi:10.1002/ecy.3163)
Supplement: Supplementary file 1 — Appendix S1 [file ECY-101-e03163-s001.pdf]

Supporting Information. Beaudrot, L., M.S. Palmer, T.M. Anderson, and C. Packer. 2020. Mixed-species groups of Serengeti grazers: a test of the stress gradient hypothesis. Ecology.

Appendix S1

Figure S1. Temporal variation in Serengeti grazers. Plots show the number of observations of six species of Serengeti grazers for each 16-day NDVI sampling bin.

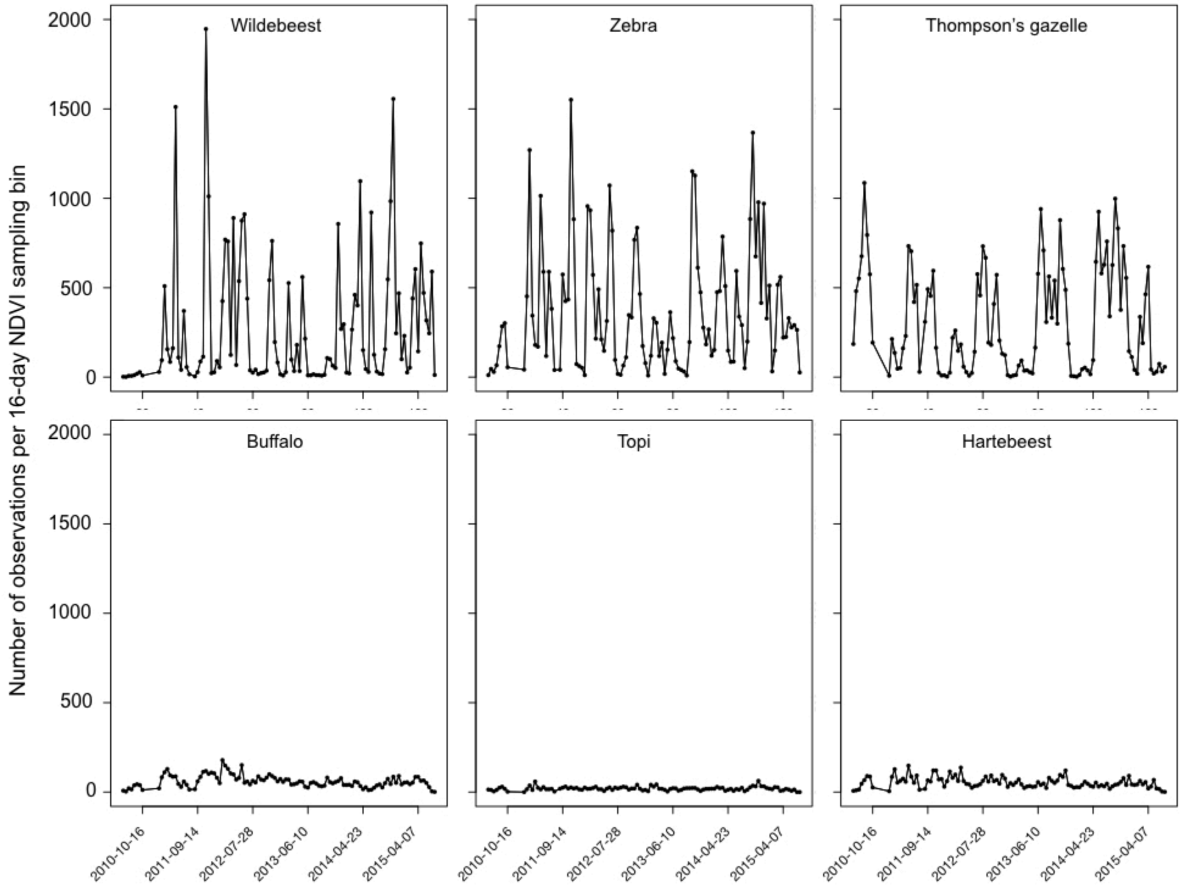

Figure S2. Autocorrelation function (ACF) plot of the proportion of camera trap observations with mixed-species groups for each 16-day NDVI sampling bin.

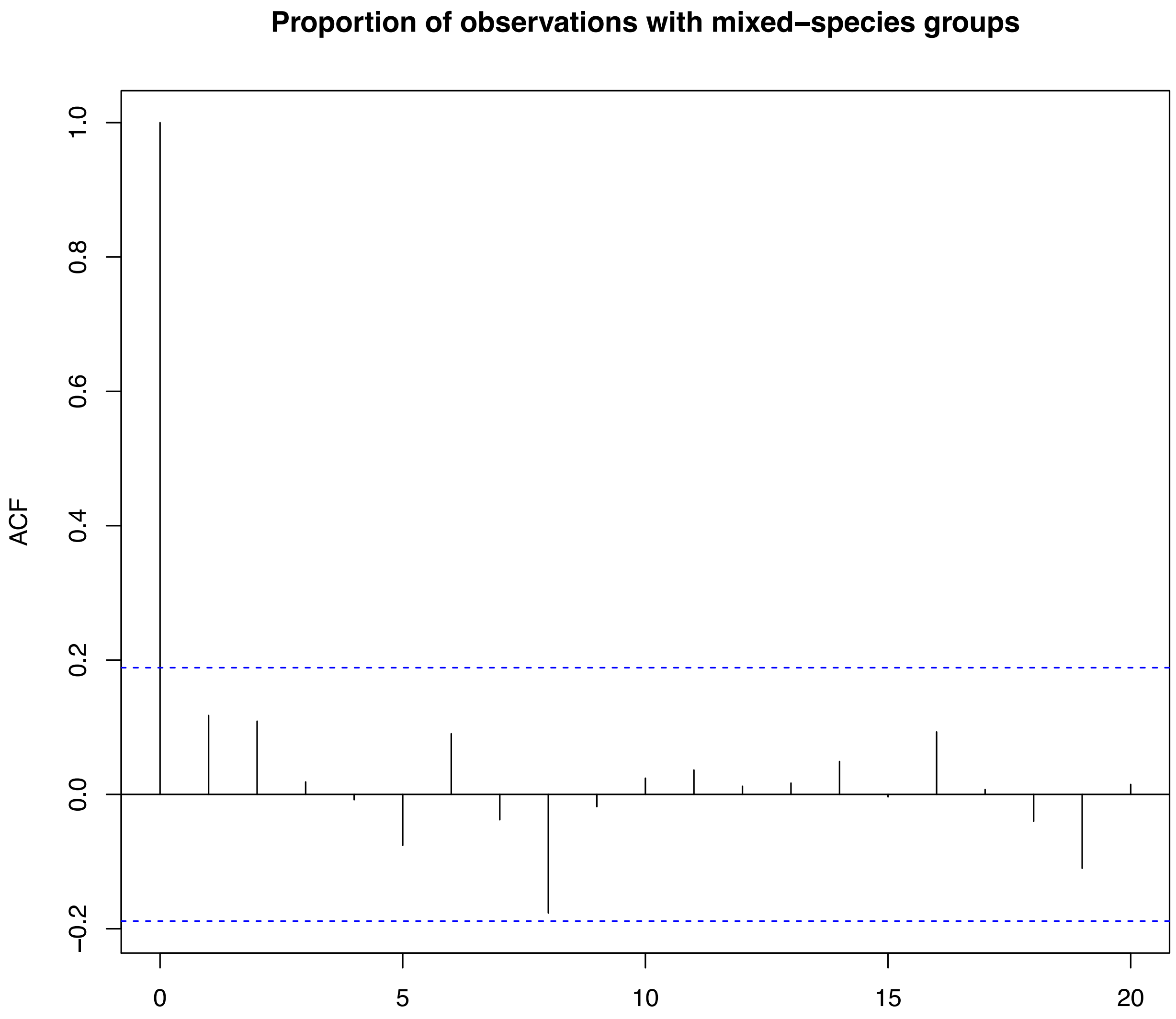

Figure S3. Boxplots illustrating relationships between predictors of mixed-species groups: **a**) distances from camera traps to nearest kopjes did not vary significantly between plains and woodland habitats **b**) lion density was significantly higher in plains habitat during the wet season, but **c**) lion density did not vary significantly between plains and woodland habitats during the dry season. **d)** Significantly more grazers were observed during dry seasons, and **e**) NDVI was significantly higher during the wet season. Pearson's correlations between continuous predictor variables were as follows: $r = 0.418$ for the correlation between wet season and dry season lion density, $r = -0.39$ for the correlation between dry season lion density and distance from nearest kopje, $r = -0.29$ for the correlation between wet season lion density and distance from nearest kopje.

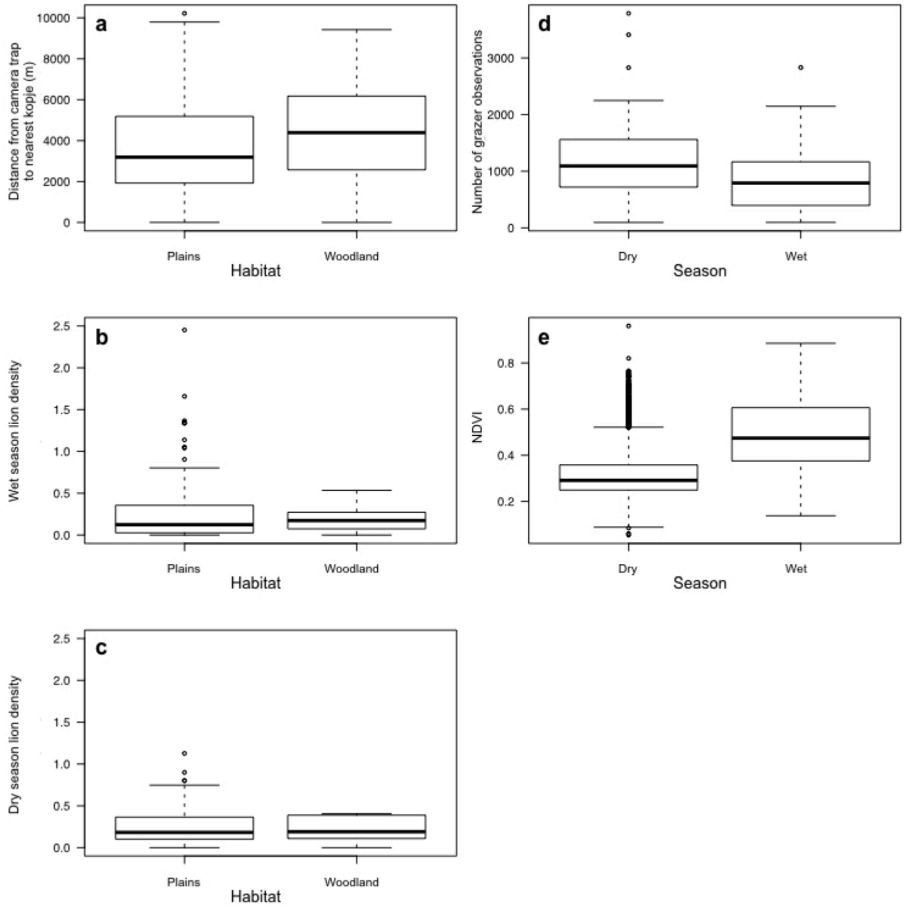

Figure S4. **Temporal variation in NDVI and Serengeti grazers excluding nocturnal observations**. For each 16-day NDVI measurement shown in green shading, the number of single-species observations are indicated by black triangles and the number of mixed species observations are indicated by blue points. Gray shading represents the wet season. The white solid line illustrates the proportion of observations of mixed-species groups. Note that the white solid line is axis-free.

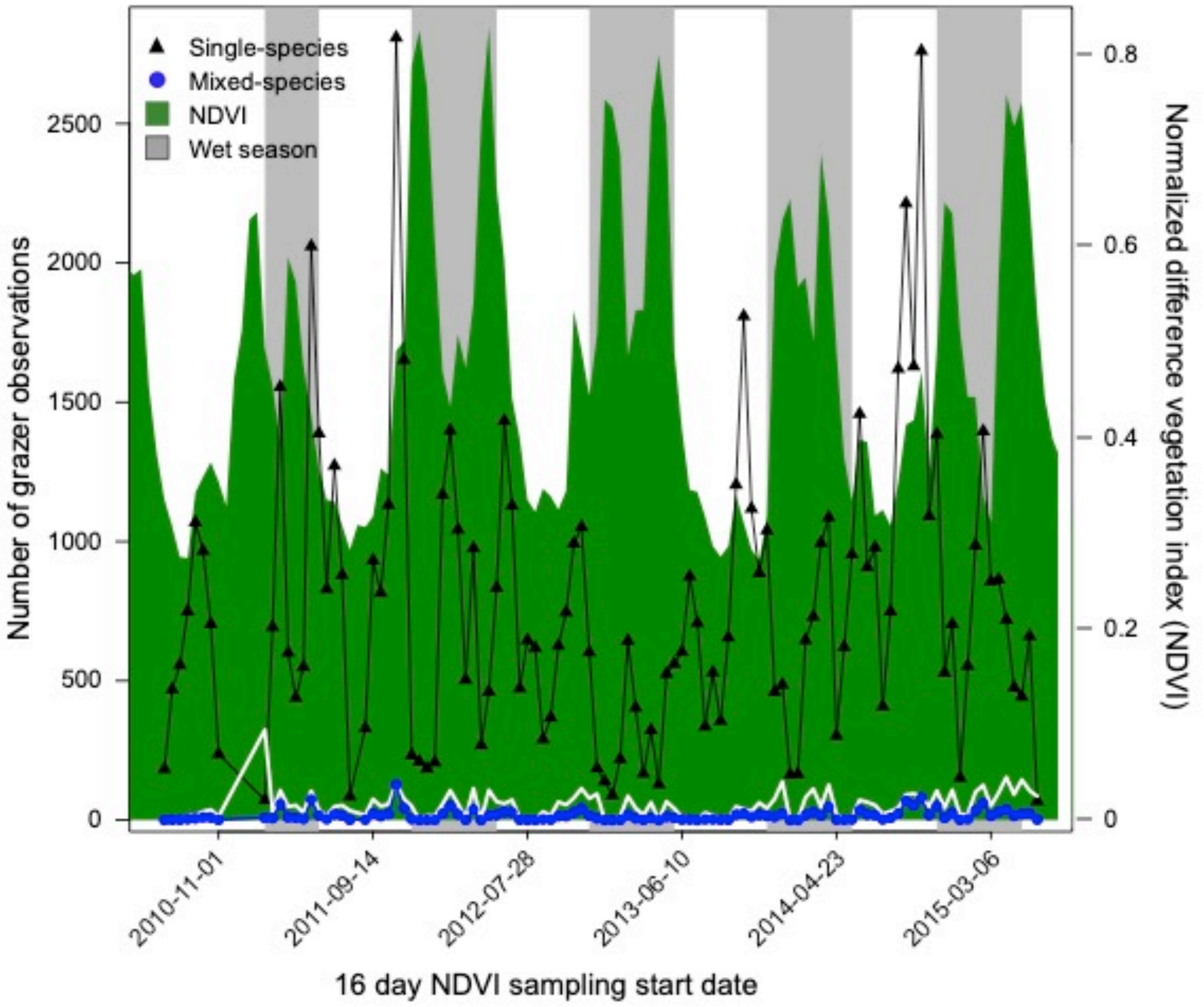

Figure S5. Model results for predictors of mixed-species groups excluding nocturnal observations. The probability of a mixed-species group occurring was significantly higher in woodland habitat, when NDVI was high, and during the wet season. The probability of a mixed-species group occurring increased as distance to kopjes declined (i.e., mixed-species groups occurred more often closer to rocky viewsheds). The plot of standardized coefficients depicts the fixed effect terms from the generalized linear mixed model predicting the occurrence of mixed-species groups. Points indicate estimates and lines indicate standard errors. Odds ratios greater than one are positive effects whereas odds ratios less than one are negative effects.

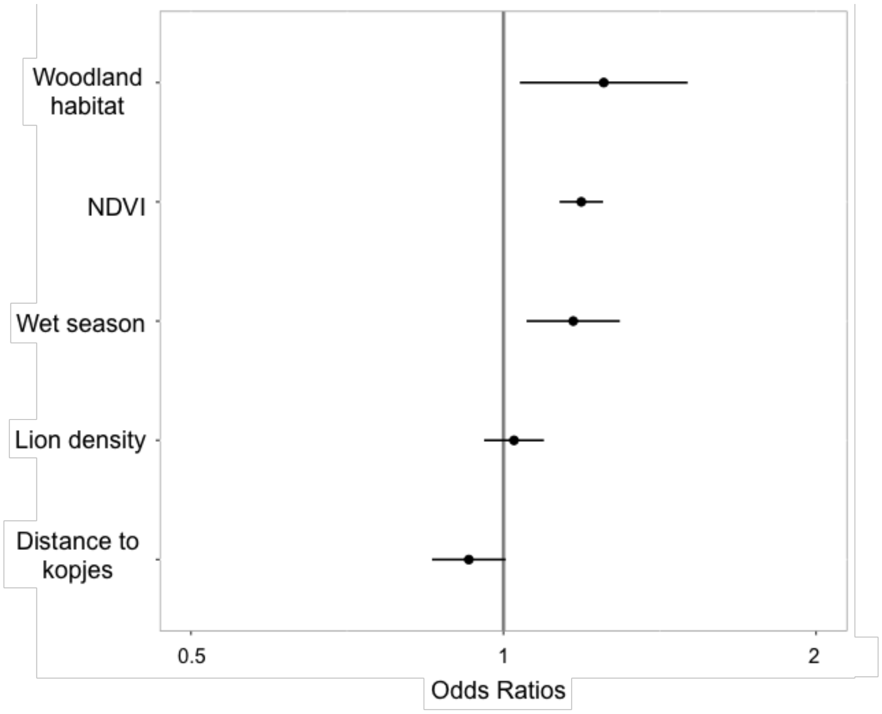

Table S1. The number of observations of each focal species individually, in a mixed-species groups, and for each species pair excluding nocturnal observations. The number of observations of mixed-species groups for each species on the left side of the table is one half of the sum for that species for its number of mixed-species groups on the right side of the table. The right side of the table counts each mixed-species group twice because it counts a mixed-species group from the perspective of each species.

	Number of observations			Number of mixed species groups for each pair of species				
Common Name	Single species	Mixed species	% mixed species	Buffalo	Thomson's gazelle	Hartebeest	Topi	Wildebeest
Buffalo	4411	16.5	0.37					
Thomson's gazelle	25475	198.5	0.78	4				
Hartebeest	4464	62.5	1.40	3	50			
Topi	1625	22.5	1.38	2	18	0		
Wildebeest	20602	693.5	3.37	4	81	9	5	
Zebra	26651	817.5	3.07	20	244	63	20	1288

Section S1

To test for potential bias from shorter sighting distances in nocturnal images because the camera trap's field of view may have been truncated during periods of darkness, we removed nighttime images from the analyses presented in the main text and present below the results based only on daytime images. Conservatively, all observations after 7:00 pm and before 6:00 am local time were excluded. Additional analyses based on daytime images demonstrate that main text results and conclusions were consistent with analyses including nocturnal observations and therefore were not biased by the inclusion of nocturnal images.

We analyzed 85,039 unique camera trap observations of Serengeti grazers during daylight hours from 28,639 camera trap sampling days between July 2010 and July 2015. Of the total observations, 83,228 (97.9%) were observations of a single species. Zebra, Thomson's gazelle and wildebeest were the most commonly observed species with more than 20,000 single-species observations each, buffalo and hartebeest had fewer than 4,500 single-species observations each and topi were observed alone on 1,625 occasions (Appendix 1: Table S1).

Camera traps recorded 1,811 occurrences of mixed-species groups. The number of mixed-species groups varied among species pairs (Appendix 1: Table S1) and over time (Appendix 1: Fig. S4). The most commonly observed mixed-species groups were of zebra and wildebeest (N=1,288 observations) while at the other extreme, topi and hartebeest were never observed together. Log-likelihood ratio (G-tests) of independence showed that the number of mixed-species observations differed significantly from the proportion expected based on the number of single-species observations for all six species ($G = 1790$, $df = 5$, $p < 0.001$), for the three migratory species ($G = 1536$, $df =$

2, $p < 0.001$), and for the three resident species ($G = 67.12$, $df = 2$, $p < 0.001$), which suggests that the mixed-species groups did not occur based on chance alone.

We used a generalized linear mixed-effects model to test the effects of predation risk and food availability on the probability of mixed-species groups in Serengeti grazers (Appendix 1: Fig. S5). Mixed-species groups were 1.25 times more likely to occur in woodland habitats than in the plains (Estimate = 0.222, SE = 0.095, $p = 0.019$) and 1.08 times less likely to occur for each unit increase in distance from kopjes (Estimate = -0.077, SE = 0.041, $p = 0.064$). They were 1.19 times more likely to occur for each unit increase in NDVI (Estimate = 0.171, SE = 0.025, $p < 0.001$) and 1.17 times more likely to occur during the wet season (Estimate = 0.155, SE = 0.053, $p = 0.003$).

Because kopjes are not distributed randomly throughout the camera trap sampling area and the increased sighting distance that kopjes provide to lions is limited, we repeated the generalized linear mixed-effects model using the subset of observations within 1000 m from a kopje. This subset of data included 16,595 total observations from 30 camera traps with 347 observations of mixed-species groups. Consistent with the complete dataset, mixed-species groups were 1.24 times less likely to occur for each unit increase in distance from kopjes (Estimate = -0.217, SE = 0.115, $p = 0.060$) and were 1.21 times more likely to occur for each unit increase in NDVI (Estimate = 0.189, SE = 0.057, $p < 0.001$).
